# Increased plasma lipid levels exacerbate muscle pathology in the mdx mouse model of Duchenne muscular dystrophy

**DOI:** 10.1186/s13395-017-0135-9

**Published:** 2017-09-12

**Authors:** Nadia Milad, Zoe White, Arash Y. Tehrani, Stephanie Sellers, Fabio M.V. Rossi, Pascal Bernatchez

**Affiliations:** 1 Department of Anaesthesiology, Pharmacology & Therapeutics, University of British Columbia (UBC), 217-2176 Health Sciences Mall, Vancouver, British Columbia V6T 1Z3 Canada; 2 Centre for Heart and Lung Innovation, St. Paul’s Hospital, 1081 Burrard Street, Rm 166, Vancouver, British Columbia Canada; 3 Department of Medical Genetics, Centre for Biomedical Research, University of British Columbia, 2222 Health Sciences Mall, Vancouver, British Columbia Canada

**Keywords:** Duchenne muscular dystrophy, Vascular disease, Lipids, Dystrophin, Apolipoprotein E, Atherosclerosis

## Abstract

**Background:**

Duchenne muscular dystrophy (DMD) is caused by loss of dystrophin expression and leads to severe ambulatory and cardiac function decline. However, the dystrophin-deficient *mdx* murine model of DMD only develops a very mild form of the disease. Our group and others have shown vascular abnormalities in animal models of MD, a likely consequence of the fact that blood vessels express the same dystrophin-associated glycoprotein complex (DGC) proteins as skeletal muscles.

**Methods:**

To test the blood vessel contribution to muscle damage in DMD, *mdx* ^*4cv*^ mice were given elevated lipid levels via apolipoprotein E (ApoE) gene knockout combined with normal chow or lipid-rich Western diets. Ambulatory function and heart function (via echocardiogram) were assessed at 4 and 7 months of age. After sacrifice, muscle histology and aortic staining were used to assess muscle pathology and atherosclerosis development, respectively. Plasma levels of total cholesterol, high-density lipoprotein (HDL), triglycerides, and creatine kinase (CK) were also measured.

**Results:**

Although there was an increase in left ventricular heart volume in *mdx*-ApoE mice compared to that in *mdx* mice, parameters of heart function were not affected. Compared with wild-type and ApoE-null, only *mdx*-ApoE KO mice showed significant ambulatory dysfunction. Despite no significant difference in plasma CK, histological analyses revealed that elevated plasma lipids in chow- and Western diet-fed *mdx*-ApoE mice was associated with severe exacerbation of muscle pathology compared to *mdx* mice: significant increase in myofiber damage and fibrofatty replacement in the gastrocnemius and triceps brachii muscles, more reminiscent of human DMD pathology. Finally, although both ApoE and *mdx*-ApoE groups displayed increased plasma lipids, *mdx*-ApoE exhibited atherosclerotic plaque deposition equal to or less than that of ApoE mice.

**Conclusions:**

Since others have shown that lipid abnormalities correlate with DMD severity, our data suggest that plasma lipids could be primary contributors to human DMD severity and that the notoriously mild phenotype of *mdx* mice might be attributable in part to their endogenously low plasma lipid profiles. Hence, DMD patients may benefit from lipid-lowering and vascular-targeted therapies.

**Electronic supplementary material:**

The online version of this article (10.1186/s13395-017-0135-9) contains supplementary material, which is available to authorized users.

## Background

Dystrophin is a 427 kDa protein encoded on the short arm of the X chromosome. It is a key member of the dystrophin-glycoprotein complex (DGC), which spans the plasma membrane and links the extracellular matrix to the intracellular cytoskeleton [1]. Dystrophin binds to cytoskeletal actin filaments and to other DGC proteins in the plasma membrane, creating an attachment point required for the maintenance of sarcolemmal structure and protection from mechanical stress [2]. Loss of functional dystrophin expression leads to development of Duchenne (DMD) and Becker muscular dystrophies (BMD) characterized by severe and progressive muscle weakness and wasting due to increased sarcolemma fragility and susceptibility to myofiber injury [2–4].

DMD is the most common type of MD, occurring in nearly 1 in 3600–6000 male births worldwide and accounting for approximately 80% of all MD cases [5, 6]. Children usually present with abnormal gait, progressive muscle weakness, calf pseudohypertrophy, and elevated serum creatine kinase (CK) levels. However, muscle hypertrophy gradually gives way to significant muscle degeneration and fibrofatty remodeling [7–9], leaving 90% of boys confined to a wheelchair by age 12 [10]. Although DMD patients exhibit devastating skeletal muscle pathology, cardiomyocytes also show increased susceptibility to mechanical stress-induced injury leading to increased heart fibrosis [11] and development of often lethal cardiac dysfunction, usually manifested as dilated cardiomyopathy (DCM) [12–15]. As a result of the severe cardiac pathology, in addition to impaired respiratory function due to significant diaphragm damage, DMD patient life expectancy is shortened to approximately 30 years of age [10, 16].

The *mdx* murine model is the most commonly used model for studying DMD; however, despite the lack of significant dystrophin expression, *mdx* mice only display mild muscle pathology. Between 2 and 8 weeks of age, *mdx* mice undergo cycles of damage, with muscle necrosis reaching up to 80% in some muscles, followed by robust myofiber regeneration [17–19]. By adulthood, *mdx* mice exhibit minimal fibrofatty infiltration and myofiber necrosis (<5% of muscle area) in most skeletal muscles [19, 20], while other muscles, such as the tongue, diaphragm, and gastrocnemius, can develop mild pathology in elderly *mdx* mice [21–23]. Cardiac muscle is also moderately affected in *mdx* mice, displaying slight fibrosis and inflammatory cell infiltration [18], although changes in heart function can only be seen in very old *mdx* mice [24] or when challenged with a dobutamine stress echocardiogram [25, 26]. Overall, the disparity between disease severity in human DMD and currently available models [18, 22] speaks to a need for more representative DMD models.

Since we and others have documented expression of DGC-associated proteins in vascular endothelial cells and smooth muscle cells [27, 28], we hypothesized that mutations of DGC proteins in the vasculature may affect the primary muscle dysfunction in MD. To test this hypothesis, *mdx* mice were given increased plasma lipid levels associated with vascular disease and atherosclerosis. This was achieved via inactivation of the apolipoprotein E (ApoE) gene, which causes accumulation of cholesterol-rich particles in the plasma [29, 30]. The resultant *mdx*-ApoE mice display increased lipid levels accompanied by significant exacerbation of the muscle pathology, better recapitulating the DMD patient phenotype. Our data suggest that the notoriously mild phenotype of *mdx* mice might be attributable to their natively low plasma lipid profiles and superior vascular health compared to humans. Since recent reports have uncovered plasma and tissue lipid abnormalities in DMD patients [31, 32], we propose that plasma lipids could be a significant contributor to MD pathology and that DMD patients may benefit from lipid-lowering and vascular-targeted therapies.

## Methods

### Animal breeding and husbandry

All animals were housed in a 12-h light/dark cycle, temperature-regulated facility, and all experiments were performed with the approval of UBC Animal Care Committee. Experimental mice were placed on regular chow (LabDiet 5001) or Western diet (Harlan, TD88137; 0.2% total cholesterol, 21% total fat, and 34% sucrose by weight) at 8 weeks of age. Mice were sacrificed at 4 and 7 months of age while under terminal anesthesia (3.5% *v*/*v* isoflurane at 2 L O_2_) via cardiac puncture and perfused with warmed Krebs solution [118 mmol/L NaCl, 22.5 mmol/L NaHCO_3_, 4 mmol/L KCl, 1.2 mmol/L NaH_2_PO_4_, 2 mmol/L CaCl_2_, 2 mmol/L MgCl_2_, and 11 mmol/L dextrose].

Experimental mice were generated using *mdx4* ^*cv*^ mice crossed with ApoE^−/−^ mice, both strains are C57BL/6 background and were acquired from Jackson Laboratories (Bar Harbor, ME). From this cross, *mdx* ^+/−^ ApoE^+/−^ littermates were bred to generate several genotypes including *mdx* ^−/−^ ApoE^+/−^ females and *mdx* ^−/y^ ApoE^+/−^ males, which were then crossed to generate two experimental groups: *mdx* ^−/−^ ApoE^+/+^ and *mdx* ^−/−^ ApoE^−/−^ (simplified as *mdx* and *mdx-*ApoE, respectively). Separately, ApoE^−/−^ mice were crossed with C57BL/6 mice to generate ApoE^+/−^ littermates. These heterozygous littermates were crossed to produce the other two experimental groups: ApoE^+/+^ and ApoE^−/−^, simplified as wild-type (WT) and ApoE, respectively. Ear clip DNA was extracted using hot NaOH and Tris-HCl extraction method [33]. Mice were genotyped using the *mdx* ^*4cv*^ primer competition method [34], and the ApoE protocol suggested by Jackson Laboratories. Mice were fed a Western diet starting at 8 weeks of age until sacrifice at 4 and 7 months. Both male and female offspring were used in this study with *n* values in each group as follows: Chow–WT (*n* = 1M, 2F), ApoE (*n* = 3M, 2F), mdx (*n* = 4M, 6F), mdx-ApoE (*n* = 6M, 7F); Western–WT 4 m (*n* = 2M, 2F), 7 m (5M, 4F); ApoE 4 m (*n* = 2M, 1F), 7 m (*n* = 5M, 4F); mdx 4 m (*n* = 3M, 4F), 7 m (*n* = 4M, 3F); and mdx-ApoE 4 m (*n* = 2M, 4F), 7 m (*n* = 6M, 4F). Stratification of data by gender at 7 months of age on Western diet revealed little to no significant difference in muscle or heart measurements (Additional file 1: Figure S8).

### Ambulatory function assessment

Step length was measured by painting the hind-feet of mice and by allowing them to run down a small corridor lined with paper approximately 1.5 m in length. Distance between steps of the same foot were measured and averaged over three consecutive runs, excluding areas of stoppage.

### In vivo heart function

Mice were anesthetized using 0.75–1.25% *v*/*v* isoflurane at 2 L O_2_ with heart rate kept between 400 and 425 BPM and a four-chamber echocardiogram was performed using the VisualSonics Vevo 2100 system. Heart chamber and ventricle wall tracings were analyzed to determine several physiological and functional cardiac parameters such as cardiac output, fractional shortening, stroke volume, ejection fraction, as well as left ventricular diameter and volume in both diastole and systole.

### Evan’s blue dye assay

As per previously elucidated protocols [35], under 3.0% isoflurane *v*/*v*, mice were injected intravenously with 200 μL of 0.5% Evan’s blue dye in PBS solution into the inferior vena cava. Animals were perfused for 10 min before sacrifice by cervical dislocation was performed and muscles were collected. After 24 h of drying at 56 °C, dried muscles were weighed and placed in 500 μL formamide for 24 h at 56 °C. Samples were spun down, 200 μL of each sample was added to 96-well plates in duplicate, and absorbance at 500 nm was measured. Standard curve was used to extrapolate ng of Evan’s blue dye which was then normalized to dry muscle tissue weight.

### Muscle histology

Diaphragm, tibialis anterior (TA), extensor digitorum longus (EDL), gastrocnemius, quadriceps femoris, and triceps brachii muscles were collected. Tissue was fixed in 10% formalin for 24 h then was transferred to 70% ethanol. Muscle was paraffin-embedded and was sectioned into 8-μm-thick slides, which were then stained with hematoxylin and eosin (H&E) or Masson’s trichrome stains. Alizarin red stain was also performed on select skeletal muscle slides to visualize calcium deposition. Percentage of fat was calculated by tracing adipocyte regions in Aperio ImageScope software and by dividing the area by the total area of the muscle. To determine percentage of muscle area occupied by healthy myofibers, non-myofiber areas (e.g., adipocytes, cholesterol accumulations, calcification, and inflammatory cells) as well as damaged, necrotic regions were subtracted from total muscle area. Collagen content was measured using a positive pixel count algorithm in Aperio ImageScope software on Masson’s trichrome stained slides using the following parameters: hue value of 0.66 and hue width of 0.25. Hearts were also fixed in 10% formalin, paraffin-embedded and stained with Masson’s trichrome.

Contralateral muscles were harvested and immediately tragacanth-embedded in cross-section, flash frozen in liquid-nitrogen-cooled isopentane and stored at − 80 °C. Frozen muscles were sectioned at − 20 °C at 8 μm in thickness and were stained with oil red O to visualized neutral lipids in muscle tissue following the protocol developed by Mehlem, Hagberg [36]. To visualize vascular density, CD31 (PECAM-1) was detected in muscle cryosections using anti-CD31 antibody (1:100 dilution, Cell Signaling, D8V9E #77699) via immunohistochemistry (IHC) protocol suggested by the manufacturer.

### Plasma analysis

Plasma was collected in heparinized tubes via cardiac puncture of unfasted mice, was spun down at 4000 RPM for 10 min at 4 °C, and was stored at − 80 °C. Plasma samples were assessed using the Siemens Advia 1800 system for levels of creatine kinase (CK NAC), cholesterol (CHOL-2), high-density lipoprotein (D-HDL), and triglycerides (TRIG-2), and assays were performed following manufacturer’s instructions.

### Aortic atherosclerosis

Hearts were cut along the atrioventricular plane and were frozen in optimal cutting temperature (OCT) compound, and aortic root sections were stained with oil red O [36]. Plaque area in the aortic root was measured in square millimeter. Whole aortas were fixed in 10% formalin, were rinsed with 70% ethanol, and were soaked in filtered Sudan IV solution (5 g Sudan IV in 500 mL 70% ethanol and 500 mL acetone) for 20 min. After aortas were rinsed in 70% ethanol, they were soaked in 80% ethanol for 20 min, then were rinsed in running water for 1 h, and were stored in 10% formalin. Photographs of pinned open aortas were taken for analysis, and percentage of plaque coverage was determined by dividing plaque area by total vessel wall area.

### Statistical analysis

Statistical analyses were performed using GraphPad Prism 6. For comparison of multiple groups at one time-point, one-way analysis of variance (ANOVA) multiple comparisons test was used to compare the means of each group. When multiple groups were compared at several time-points, a two-way ANOVA multiple comparisons test was used. Tukey’s method was used to correct for multiple comparisons. A *P* value of less than 0.05 was considered statistically significant. Figures show data as mean plus standard error of the mean (SEM) and *n* indicates the number of mice per experiment.

## Results

### Increased plasma lipid levels cause minor cardiac changes in *mdx* mice

**Fig. 1:**
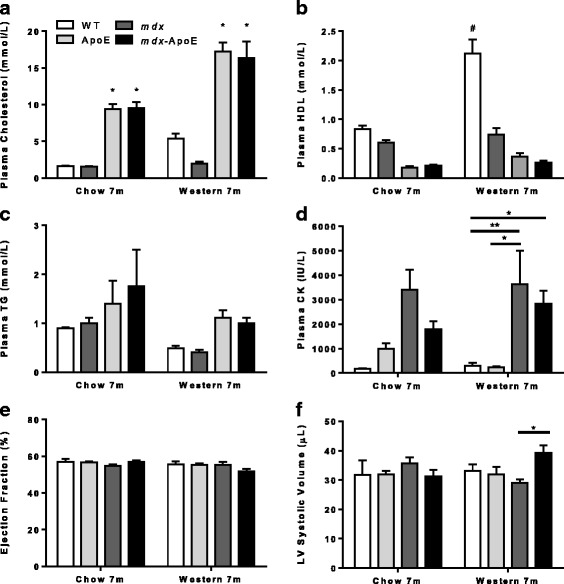
Plasma lipid and creatine kinase levels and cardiac parameter on chow and Western diets. Plasma total cholesterol (**a**), HDL (**b**), triglycerides (**c**), and creatine kinase (**d**) as well as cardiac parameters from echocardiogram at 7 months of age on chow and Western diet: ejection fraction (**e**) and left ventricular systolic volume (**f**). Chow: WT (*n* = 2–3), ApoE (*n* = 5), *mdx* (*n* = 6–7), *mdx*-ApoE (*n* = 8–11). Western: WT (*n* = 6–9), ApoE (*n* = 4–7), *mdx* (*n* = 6), *mdx*-ApoE (*n* = 8–11). Mean + SEM. (**a**–**c**) *P < 0.05 compared to WT and ApoE #*P* < 0.05 compared to all other groups; (**d**–**f**) **P < 0.01

Hyperlipidemia in *mdx* mice did not result in lethality or complete loss of ambulatory function. As expected, total plasma cholesterol was elevated in ApoE and *mdx*-ApoE groups compared to WT and *mdx* mice fed a normal chow diet (Fig. 1a), confirming functional loss of ApoE. Total cholesterol was further elevated when ApoE and *mdx*-ApoE mice were fed a Western, atherogenic diet (Fig. 1a). WT and *mdx* mice displayed low total cholesterol in both chow- or Western diet-fed groups, providing further evidence that mice with normal ApoE expression have endogenously non-atherogenic lipid profiles even on a lipid-rich diet. This was accompanied by the expected raise in atheroprotective high-density lipoprotein (HDL) in WT mice fed a Western diet (Fig. 1b). However, *mdx* mice did not show a significant increase in plasma HDL on a Western diet (Fig. 1b), lending credence to observations that DMD patients show abnormal lipid profiles [31, 32, 37]. Surprisingly, no significant differences were observed in plasma TG levels for Western- and chow-fed groups (Fig. 1c). Although studies have shown that levels of plasma CK, a marker of muscle damage, start to drop in *mdx* mice after 23 weeks of age [38], *mdx* and *mdx*-ApoE strains on Western diet showed similarly robust increases in plasma CK levels at 7 months of age, while this trend was not significant in chow-fed groups (Fig. 1d). Similar plasma CK and lipids were observed at 4 months of age on Western diet (Additional file 2: Figure S1A–D).

Using echocardiography, parameters of heart function were monitored in vivo at 4 and 7 months of age since DMD patients develop severe DCM. Although, we observed a significant increase in left ventricular (LV) systolic volume, indicating mild ventricle dilation in *mdx*-ApoE mice compared to *mdx* at 7 months when fed a Western diet (Fig. 1f). However, this was not significantly different from WT and ApoE controls, and there was no change in heart function parameters such as ejection fraction (Fig. 1e). Similarly, no significant changes were observed in cardiac output, fractional shortening, stroke volume, left ventricular diameter in diastole and systole of chow- or Western diet-fed groups (Additional file 3: Figure S2A–F) and no significant changes were observed in any cardiac parameters at 4-month time-point for both chow- and Western diet-fed groups (data not shown). On chow diet, there was no significant difference in animal body weight between 3 and 7 months of age; however, Western diet-fed wild-type animals showed significantly increased body weight by 6 and 7 months of age compared to ApoE and *mdx*-ApoE groups, respectively (Additional file 4: Figure S3A–B). Heart weights and heart to body weight ratios were also similar between groups at 7 months on Western diet (Additional file 4: Figure S3C–D). Cardiac function and weight were stratified by gender, and only WT heart weights were significantly different between males and females at 7 months of age on Western diet (Additional file 1: Figure S8). Although left ventricular systolic volume was significantly larger in *mdx*-ApoE mice compared to *mdx* mice at 7 months of age on Wester diet, histological observation of cardiac muscle revealed similarly minor cardiac fibrosis in both *mdx* and *mdx*-ApoE mice compared to controls by 7 months of age on Western diet (Additional file 4: Figure S3E).

### Hyperlipidemic *mdx* mice display impaired ambulatory function and sustained hindlimb hypertrophy

**Fig. 2:**
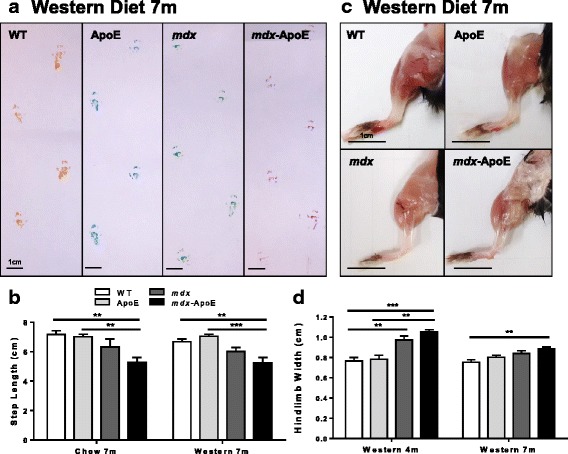
Stride length measurements and hindlimb hypertrophy on chow and Western diets. Representative images of Western diet-fed group gait-tracking at 7 months of age, scale bar 1 cm (**a**) and quantification of stride length in centimeter on chow and Western diets (**b**). Representative images of Western diet-fed hindlimbs at 7 months of age, scale bar 1 cm (c) and quantification of hindlimb width at 4 and 7 months on Western diet. Chow: WT (*n* = 3), ApoE (*n* = 5), *mdx* (*n* = 3), and *mdx*-ApoE (*n* = 9). Western: WT 4 m (*n* = 4), 7 m (*n* = 6); ApoE 4 m (*n* = 2), 7 m (*n* = 7–8); *mdx* 4 m (*n*=)7, (*n* = 5–7); and *mdx*-ApoE 4 m (*n* = 5), 7 m (*n* = 10). Mean + SEM. *P < 0.05 **P < 0.01 ***P < 0.001 ****P < 0.0001

In terms of ambulation, we saw noticeable impairment in the walking ability of hyperlipidemic *mdx* mice. Compared to WT or ApoE mice, stride length measurements revealed a significant 20–25% decrease in stride length in the *mdx*-ApoE mice fed a chow or Western diet at 7 months of age, whereas *mdx* mice showed a non-significant reduction in stride length (Fig. 2a–b). Following euthanasia at 7 months, we noticed sustained gross skeletal muscle size increases in *mdx*-ApoE mice fed a Western diet compared to WT (Fig. 2c–d) and similar results were seen in 7-month-old groups fed chow (data not shown). Since calf hypertrophy is a relatively early event in DMD, we performed similar analyses in a second cohort at an earlier 4-month time-point and noticed even more severe hindlimb hypertrophy in *mdx* and *mdx*-ApoE mice fed a Western diet, with no significant difference between dystrophin-deficient groups (Fig. 2d).

### Elevated plasma lipid levels exacerbate *mdx*-associated myofiber damage and skeletal muscle remodeling

**Fig. 3:**
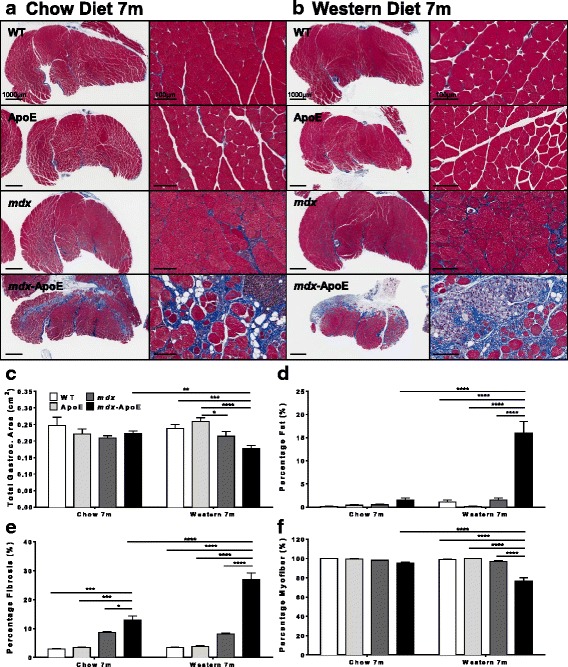
Gastrocnemius muscle size and composition at 7 months on chow and Western diets. Representative images of gastrocnemius from 7-month-old mice stained with Masson’s trichrome, scale bars 1000 μm (left) and 100 μm (right), on chow (**a**) and on Western diet (**b**). Quantification of total gastrocnemius area in square centimeter (**c**) and percentage of area composed of fat (**d**), fibrosis (**e**), and healthy myofiber (**f**). Chow: WT (*n* = 3), ApoE (*n* = 4), *mdx* (*n* = 10); *mdx*-ApoE (*n* = 13). Western: WT (*n* = 9), ApoE (*n* = 8), *mdx* (*n* = 7), and *mdx*-ApoE (*n* = 10). Mean + SEM. *P < 0.05 **P < 0.01 ***P < 0.001 ****P < 0.0001

**Fig. 4:**
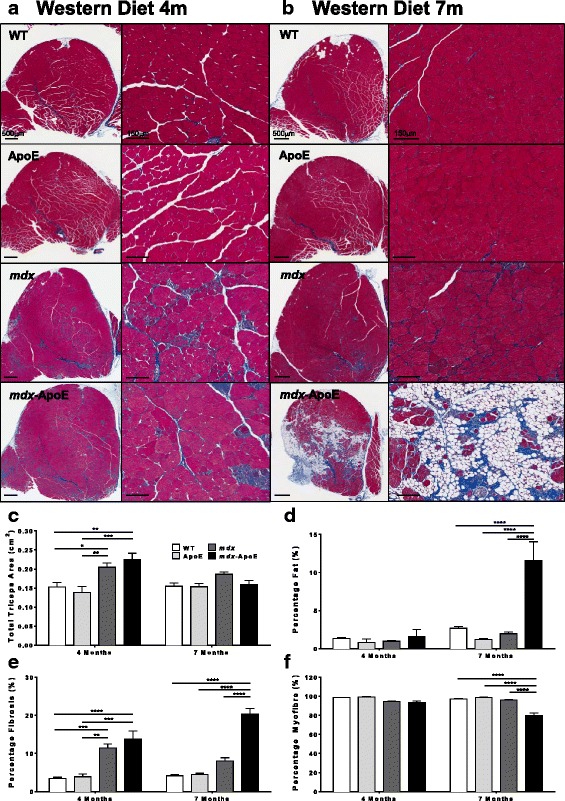
Triceps brachii muscle size and composition at 4 and 7 months on Western diet. Representative images of triceps brachii stained with Masson’s trichrome, scale bars 500 μm (left) and 150 μm (right) at 4 (**a**) and 7 months of age (**b**) on Western diet. Quantification of total triceps brachii area in square centimeter (**c**) and percentage of area composed of fat (**d**), fibrosis (**e**), and healthy myofiber (**f**). WT 4 m (*n* = 4), 7 m (*n* = 9); ApoE 4 m (*n* = 3), 7 m (*n* = 9); *mdx* 4 m (*n* = 7), 7 m (*n* = 7); *mdx*-ApoE 4 m (*n* = 6), 7 m (*n* = 10). Mean + SEM. *P < 0.05 **P < 0.01 ***P < 0.001 ****P < 0.0001

Muscles isolated from WT and ApoE mice showed normal histological features in all skeletal muscle stained with Masson’s trichrome (Figs. 3 and 4) and H&E (not shown) when fed a chow or Western diet. However, some muscles exhibited severely worsened myofiber damage in *mdx-*ApoE mice compared to *mdx* mice with natively lower plasma lipids, such as gastrocnemius (Fig. 3a–b). Results were stratified based on gender, and no significant difference between males and females was observed, as seen in Additional file 1: Figure S8. For example, when fed a Western diet, total gastrocnemius muscle area from 7-month-old *mdx*-ApoE mice was significantly decreased compared to WT and ApoE controls (Fig. 3c). In addition, *mdx*-ApoE gastrocnemius muscles showed significant changes in muscle composition compared to all other groups, such as drastically increased fat and collagen infiltration (approximately 15 and 25% of total muscle area, respectively) as well as decreased healthy myofiber area (Fig. 3d–f). Early signs of muscle damage and fibrosis can be observed in *mdx*-ApoE gastrocnemius by 4 months of age on a Western diet, with significant increases in fibrosis and a mild increase in fat deposition (Additional file 5: Figure S4A–D). Chow-fed *mdx*-ApoE gastrocnemius muscles at 7 months of age showed more modest but noticeable pathology (Fig. 3a), with significantly less muscle atrophy, fat infiltration and collagen deposition as well as myofiber necrosis compared to the Western diet-fed *mdx*-ApoE mice (Fig. 3c–f).

Similarly, the triceps brachii muscle was severely aggravated in *mdx*-ApoE mice as seen in Fig. 4a–b. Analysis of total muscle area revealed an initial increase in total area of *mdx*-ApoE triceps brachii at 4 months on a Western diet (Fig. 4c). Although this hypertrophy evened out by 7 months of age (Fig. 4c), there were dramatic changes to muscle composition in *mdx*-ApoE triceps brachii muscles compared to all other groups at this time-point: increased fat and collagen deposition (approximately 10 and 20% of total muscle area, respectively) and decreased viable muscle area (Fig. 4d–f). Again, increased collagen deposition in both Western diet-fed *mdx* and *mdx*-ApoE triceps could be observed as early as 4 months of age (Fig. 4e).

Conversely, we observed only minor, non-significant trend towards exacerbation of muscle pathology in the quadriceps femoris of *mdx-*ApoE mice: similar increase in fibrosis between *mdx* and *mdx*-ApoE groups with no significant fat infiltration (Additional file 6: Figure S5A–E). Similarly, hyperlipidemia did not affect *mdx*-associated pathology in the diaphragm muscle (Additional file 7: Figure S6A): both *mdx* and *mdx*-ApoE mice on a Western diet showed significant levels of fibrosis and myofiber damage with similar decreases in healthy myofiber area at 4 and 7 months on Western diet (Additional file 7: Figure S6B–E). Finally, we did not observe any dramatic muscle pathology in the tibialis anterior (TA), soleus or extensor digitorum longus (EDL) muscles collected (data not shown). Together, our data suggest increased muscle atrophy, myofiber damage, and fibrofatty infiltration in some but not all *mdx*-associated muscles.

**Fig. 5:**
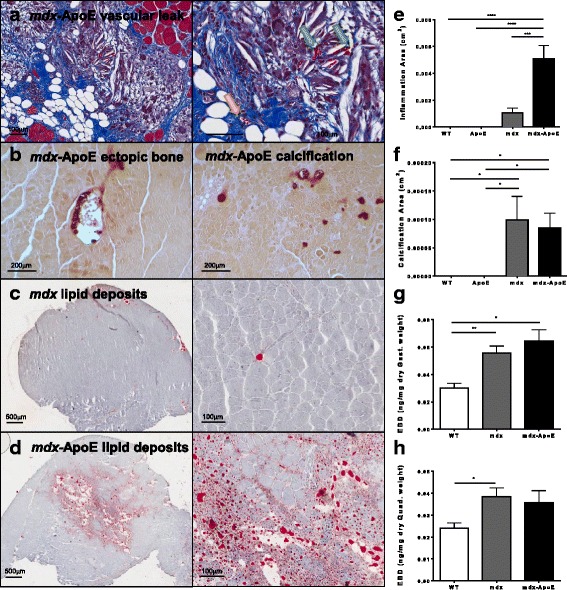
Additional skeletal muscle histological features. Example of vascular leak in area of damage within *mdx*-ApoE Masson’s trichrome stained triceps brachii (yellow arrow indicates blood vessel and green arrows indicate red blood cells in muscle tissue), scale bars 100 μm (**a**). Ectopic bone (left) and calcification (right) in 4-month-old *mdx*-ApoE gastrocnemius stained with Alizarin red, scale bars 200 μm (**b**). Lipid deposits in *mdx* (**c**) and *mdx*-ApoE (d) oil red O stained triceps brachii, scale bars 500 μm (left) and 100 μm (right). Quantification of inflammation area (**e**) and calcification area (**f**) at 7 months of age on Western diet. Quantification of Evan’s blue dye extravasation in gastrocnemius (**g**) and quadriceps femoris (**h**) muscles of chow-fed wild-type, *mdx* and *mdx*-ApoE mice. Chow: WT (*n* = 5), *mdx* (*n* = 8), and *mdx*-ApoE (*n* = 9). Western: WT (*n* = 9), ApoE (*n* = 8), *mdx* (*n* = 5), and *mdx*-ApoE (*n* = 9). Mean + SEM *P < 0.05 **P < 0.01 ***P < 0.001 ****P < 0.0001

Throughout *mdx*-ApoE muscle, regions of severe muscle damage were marked by regional fatty and fibrotic remodeling with scattered remaining muscle fibers, which is indicative of endomysial fatty infiltration, as seen in Fig. 5a of Western diet-fed *mdx*-ApoE triceps brachii. In addition, giant foam cells and structures characteristic of cholesterol clefts are clearly visible in such areas of severe damage (Fig. 5a). Interestingly, outside these regions of fibrofatty replacement, adjacent muscle tissue displays little fibrosis despite evidence of myofiber degeneration and regeneration (increased fiber size variability and centrally nucleated cells). A common sign of muscle damage and remodeling, significant areas of muscle calcification were observed in *mdx* and *mdx*-ApoE mice and occasional ectopic bone formation was seen in *mdx*-ApoE muscles, as confirmed via Alizarin red staining (Fig. 5b). However, measurement of total calcification area in 7-month-old, Western diet-fed groups revealed significant increase in calcification in both *mdx* and *mdx*-ApoE without significant difference between dystrophin-deficient groups (Fig. 5f). Moreover, lipid deposits were seen forming to a much larger extent both within myofiber cells and within adipocytes of *mdx*-ApoE muscle compared to *mdx*, as demonstrated by oil red O staining of the triceps brachii at 7 months of age on Western diet (Fig. 5c–d).

At higher magnification, severe vascular leak can be seen within damaged *mdx* (not shown) and *mdx-*ApoE muscle area (Fig. 5a, right), as evidenced by erythrocytes (green arrows) being visible in muscle tissue remote from blood vessels (yellow arrow), consistent with vascular abnormalities observed by others [28, 39, 40]. The presence of red blood cells outside blood vessels in muscle tissue was observed in 100% of gastrocnemius muscles from *mdx* and *mdx*-ApoE mice with a 0% occurrence in WT and ApoE groups. In addition, inflammatory cell infiltration was significantly increased in *mdx*-ApoE gastrocnemius muscles by 7 months of age on Western diet (Fig. 5e). Quantification of vascular permeability via intravenous injection of Evan’s blue dye revealed significant increase in dye extravasation in the gastrocnemius muscle of *mdx* and *mdx-*ApoE mice compared to WT controls by 8–10 months of age on chow diet (Fig. 5g). Even in less affected muscles, such as the quadriceps femoris, Evan’s blue dye leak was significantly increased in the *mdx* group compared to wild-type mice, although not significantly different in *mdx*-ApoE mice due to large variability (Fig. 5h). Also, areas of significant damage in both *mdx* and *mdx*-ApoE mice were shown to exhibit normal, if not increased vascular density, as visualized by H&E and CD31 (PECAM-1) stained serial sections in the triceps brachii muscle, indicating that the muscle damage observed is not the result of infarct-induced ischemia (Additional file 8: Figure S7A–B).

### Loss of dystrophin does not aggravate vascular atherosclerosis

**Fig. 6:**
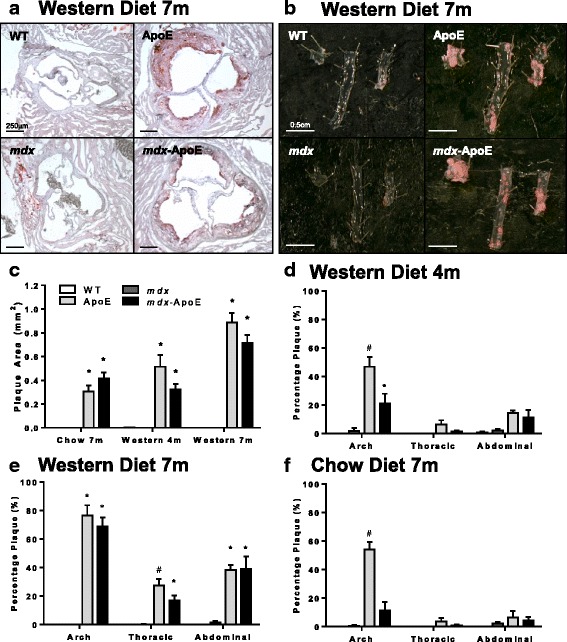
Atherosclerotic plaque accumulation in the root, arch, thoracic, and abdominal aortic segments. Representative images of plaque accumulation in Western diet-fed groups at 7 months of age in the aortic root, scale bar 250 μm (**a**) and aortic segments: arch, thoracic, and abdominal (left to right), scale bar 0.5 cm (**b**). Quantification plaque area in the aortic root (**c**) and plaque percentage in aortic segments of Western diet-fed groups at 4 (**d**) and 7 months of age (**e**), as well as chow-fed groups at 7 months (**f**). Chow: WT (*n* = 3), ApoE (*n* = 5), *mdx* (*n* = 4), and *mdx-*ApoE (*n* = 4–7). Western: WT 4 m (*n* = 3–4), 7 m (*n* = 5–7); ApoE 4 m (*n* = 4), 7 m (*n* = 5–7); *mdx* 4 m (*n* = 2–5), 7 m (*n* = 3–4); and *mdx-*ApoE 4 m (*n* = 5–6), 7 m (*n* = 5–6). Mean + SEM. *P < 0.05 compared to WT and *mdx* #*P* < 0.05 compared to all other groups

Since ApoE-null mice are a commonly used model of atherosclerosis and micro-infarcts have been suggested as causes for myofiber damage in DMD, we investigated whether loss of dystrophin expression in the vasculature of ApoE mice had any effect on atherosclerosis development—a potential confounding factor that could rationalize increased myofiber damage in *mdx*-ApoE mice. Analysis of atherosclerotic plaque load in the aortic root (Fig. 6a) by oil red O staining showed a significant increase in plaque area in ApoE and *mdx*-ApoE at 4 and 7 months on chow and Western diets; however, there was no significant difference between hyperlipidemic groups (Fig. 6c). Atherosclerotic plaque coverage in the arch, thoracic, and abdominal segments of the aorta, as quantified from Sudan IV stained whole aortas (Fig. 6b), was found to be significantly lower in *mdx*-ApoE group compared to ApoE mice in the arch at 4 months of age and in the thoracic aorta at 7 months on a Western diet (Fig. 6d–e). On chow at 7 months of age, *mdx*-ApoE mice also showed lower atherosclerotic burden than ApoE controls in the aortic arch (Fig. 6f). Together, these data demonstrate that atherosclerosis burden of *mdx*-ApoE mice is equal to or less than that of ApoE mice and therefore likely does not account for the extent of muscle damage observed in *mdx*-ApoE mice.

## Discussion

Our investigation of the effects of hyperlipidemia in the *mdx* mouse model of DMD revealed significantly worsened pathology in some key skeletal muscles by 7 months of age on chow and Western diets, with fibrotic changes observable as early as 4 months of age. In particular, gastrocnemius and triceps brachii muscles from the *mdx*-ApoE group showed increased fibrosis and fat infiltration as well as decreased healthy myofiber area compared to all other groups, while quadriceps femoris, diaphragm, and cardiac muscle damage was not further exacerbated in our double-disease model. Interestingly, the plasma CK concentration did not correlate to the level of muscle damage observed in the *mdx*-ApoE model; however, this phenomenon has been noted in late stage disease of both *mdx* mice and DMD patients where levels of CK drop dramatically over time with the loss of myofiber area [18, 41, 42]. Consistent with previous studies which showed that cardiac function is usually unaffected in *mdx* mice of this age [26, 43], our *mdx-*ApoE model only showed significant cardiac dilation compared to *mdx* mice and mild fibrosis of the heart was observed in both *mdx* and *mdx*-ApoE mice alike. Despite the effect of hyperlipidemia on skeletal muscle, the lack of dramatic exacerbation of cardiac muscle pathology in our *mdx*-ApoE model indicates that the DMD pathogenesis in these two muscle types may be different and not equally affected by increased circulating plasma lipids.

Since our data corroborate the findings of a previous study that observed blunted plaque deposition in *mdx*-ApoE mice compared to ApoE mice [44] and we have shown that there is normal or even increased vascular density in areas of muscle damage, the exacerbation of muscle pathology in *mdx*-ApoE is likely not muscle necrosis resulting from plaque-induced ischemic events. Instead, our data suggests that the increased vascular permeability observed in both *mdx* and *mdx*-ApoE muscle may facilitate the infiltration of plasma lipids into muscle tissue in the *mdx*-ApoE mice, which have high circulating cholesterol and lipids. Based on the histological features of severe muscle lesions in *mdx*-ApoE mice compared to *mdx* mice with endogenously low circulating lipids, we propose that leakage of plasma lipids from blood vessels into susceptible muscle tissues results in myofiber toxicity and that, the higher the plasma lipids are, the greater the downstream myofiber damage and fibrofatty replacement.

### *mdx*-ApoE as an improved model of DMD

The *mdx* mouse is notorious for failing to exhibit the severity of muscle damage observed in DMD patients, displaying only increased centralized nuclei, little fibrosis, early myofiber necrosis associated with robust regeneration (except in the diaphragm), slight reduction in muscle force, and inconsequential cardiac phenotypes [21]. With decreased step length and dramatic exacerbation of muscle pathology in *mdx*-ApoE mice as early as 4–7 months of age, we suggest that our model exhibits superiority to the commonly used *mdx*/*utrn* DKO model of MD in key areas. The latter model shows early onset of muscle dystrophy (degeneration, macrophage infiltration, and necrosis) and death due to double mutation of DGC gene products, dystrophin, and utrophin, which is uncharacteristic of DMD patients. In contrast, our model offers a more human-like setting of muscle damage with true fibrofatty infiltration better suited for therapeutic and pharmacological testing. While loss of ambulatory function and impairment of cardiac function were not observed in this study, it is possible that the 7-month time-point of our study was not sufficient for skeletal and cardiac muscle damage to incur major functional deficits and that studies of longer duration may lead to further pathological exacerbation. Although the gastrocnemius and triceps brachii muscles are only mildly affected in the regular *mdx* model, the pattern of muscles affected in our hyperlipidemic model are more consistent with DMD pathology [45, 46]. The mechanism by which some muscles are affected while others are relatively untouched remains a mystery. Some speculate that the pattern of muscle damage in a particular type of MD is determined by the predominant myofiber type [47]; however, DMD muscle pathology is present in both slow and fast type-rich muscles. In addition, since increased vascular leak was observed in both affected and unaffected muscles (gastrocnemius and quadriceps femoris, respectively), it seems that the pattern of muscle damage is more complex than simply through increased vascular permeability.

### Plasma lipids: a new spin on the vascular theory?

Since pre-clinical DMD biopsies show characteristic endothelial cell swelling, injury, platelet embolism, and angiogenic impairment [48, 49], some have suggested that blood vessels may contribute to myofiber degeneration—referred to as the ‘vascular hypothesis in MD.’ While *mdx* mice show abnormal angiogenesis and mechanotransduction [49, 50], lack of ischemic infarcts in human and mouse biopsies have casted doubts about the role of the vasculature in DMD [7]. Instead, we propose that the presence of lipid-rich infiltrates in patients and in our model of hyperlipidemic DMD may act as a primary effector in disease progression, as evidenced by our histological observation of high vascular density, cholesterol clefts, red blood cell extravasation, and vascular leak of Evan’s blue dye into muscle tissue. Disruption of endothelial barrier function caused by lack of dystrophin expression helps explain how muscle oedema precedes fibrofatty replacement [51], which may be facilitating the infiltration of immune cell and circulating lipids into muscle tissue. The increase in Evan’s blue dye leak from the vasculature in *mdx* and *mdx-*ApoE mice supports the idea that there is inherent vascular dysfunction associated with dystrophin-deficiency and that, in the context of hyperlipidemia, this may facilitate circulating lipids to infiltrate the muscle tissue and to cause muscle damage or affect muscle repair and remodeling. Further investigation may reveal whether infiltration of plasma contents into muscle instigates myofiber damage and degeneration through increased oxidative stress and immune reactivity and/or affect muscle signaling and stem cell differentiation, favoring fibrofatty remodeling instead of myofiber regeneration. In addition, dystrophic myofibers have previously been shown to be more susceptible to oxidative stress and free radical-mediated injury [52–55], which is markedly increased in the context of hyperlipidemia. The vast regions of adipocyte replacement and discovery of lipid-filled intramyofiber vesicles in our *mdx*-ApoE muscle suggest that lipid accumulation in muscle may be a cause as well as a consequence of severe muscle damage.

### Lipids and lipoproteins as primary mediator of DMD and potential therapeutic targets

With growing evidence suggesting significant plasma lipid-related abnormalities in MD patients [31, 32], it remains to be determined whether alterations in lipid metabolism in patients are primary or secondary to MD. Our observation that *mdx* mice on a Western diet fail to show the expected raise in HDL particles suggests liver-related abnormalities in the regulation of plasma lipoproteins levels, a key aspect of our work that warrants a more in-depth investigation in DMD patients. Triglycerides and cholesterol have been found to be elevated in DMD muscles, whereas phospholipids ratios are lowered [32] and our data implicate lipids of plasma origin in muscle degeneration. It has been shown that a lipid-rich environment may have detrimental effects on sarcolemmal integrity, since the lipid content in the plasma membrane of muscle fibers is tightly regulated [56, 57]. Moreover, many muscle-related diseases are characterized by changes in phospholipid composition and oxidation which have significant effects on membrane stability, calcium signaling, and myofiber damage [58, 59]. Although the respective contributions of various lipids (LDL, HDL, IDL, VLDL, TG, etc.) to muscle degeneration are currently unknown, oxidized-LDL is known to disrupt endothelial function and to contribute significantly to the development of vascular disease while HDL plays an atheroprotective role in the circulation [60]. Since lipid levels were elevated in the *mdx*-ApoE model by 4 months of age, before dramatic muscle damage was observed, this indicates that it is likely the cumulative exposure to high circulating lipids which leads to an exacerbated muscle phenotype and not through an acute response. The accumulation of lipids in the muscle tissue over time could have direct toxic effect on myofibers or trigger inflammation and tissue destruction, the latter mechanism being supported by our histological data.

To err on the side of caution, while our *mdx*-ApoE model more closely matches the pathology observed in DMD patients than the *mdx* mouse with its endogenously low lipid levels, some important differences remain. For instance, although ApoE mice have lipid levels more representative of the human lipid profile than wild-type mice, the levels of circulating VLDL and chylomicron remnants are higher than those typically found in humans [61]. Nevertheless, it is to be noted that the atherosclerotic lesions in ApoE mice develop spontaneously through a similar mechanism as those seen in hyperlipidemic humans [62]. In addition, our model differs from DMD patients as *mdx*-ApoE mice displayed only small changes to ambulatory function and failed to develop significant DCM, which are important pathological outcomes of DMD.

In all, our data suggest that DMD patients could benefit from diets routinely recommended to hyperlipidemic patients that attempt to raise anti-atherogenic HDL cholesterol and to lower pro-atherogenic LDL-associated cholesterol. Finally, safely modifying a DMD patient’s lipid profile with cholesterol or TG-lowering medications should also be investigated. This is supported by a previous study which found that the lipid-lowering medication simvastatin, a member of the statin family, significantly improves muscle function and fibrosis with no evidence of rhabdomyolysis in *mdx* mice [63]. However, this benefit was believed to be mediated through the drug’s antioxidant “pleiotropic” effects since *mdx* lipid levels, although endogenously low, were not further decreased with simvastatin treatment. Interestingly, many groups have shown benefits using vascular-targeted therapies in *mdx* or DMD, such as nitric oxide (NO) donors and angiotensin II receptor blockers (ARBs) [64–67]. Hence, optimization of lipid-lowering or vascular-targeted approaches in DMD could offer unique therapeutic and management options that should be investigated.

## Conclusions

In all, our study revealed mild cardiac changes, impaired ambulatory function, and dramatically worsened skeletal muscle pathology in the hyperlipidemic *mdx*-ApoE model of DMD, which better recapitulated disease severity observed in DMD patients than standard *mdx* mice. Therefore, our data suggest that circulating lipids may be significant contributors to the development and progression of DMD pathology and that murine models may exhibit more mild phenotypes due to their natively low plasma lipid levels. Since clinical research has indicated potential vascular and lipid abnormalities in DMD, it is clear that the pathophysiology of DMD is more complex than previously understood and that lipid-lowering and vascular-targeted therapies may be beneficial in DMD treatment.

## Additional files

Additional file 1: Figure S8. Muscle and cardiac data stratified at 7 months on Western diet stratified by gender. WT (*n* = 5M, 4F); ApoE (*n* = 5M, 4F); *mdx* (*n* = 4M, 3F); and *mdx*-ApoE (*n* = 6M, 4F). Mean (SEM), *P* < 0.05 in bold. (PDF 434 kb)

Additional file 2: Figure S1. Plasma lipid levels at 4 months of age on Western diet. Plasma CK in IU/L (A), total cholesterol in mmol/L (B), HDL in mmol/L (C) and TG in mmol/L (D) at 4 months of age on Western diet. WT (*n* = 4), ApoE (*n* = 4), *mdx* (*n* = 7), and *mdx*-ApoE (*n* = 5). Mean + SEM. *P < 0.05 compared to WT and *mdx* #*P* < 0.05 compared to all other groups. (PDF 158 kb)

Additional file 3: Figure S2. Heart function parameters on chow and Western diets. Percent fractional shortening (A), cardiac output in mL/min (B), left ventricular diastolic diameter (C) and systolic diameter in mm (D), left ventricular diastolic volume (E) and stroke volume in μL (F). Chow: WT (*n* = 3), ApoE (*n* = 5), *mdx* (*n* = 6), and *mdx*-ApoE (*n* = 11). Western: WT (*n* = 6), ApoE (*n* = 7), *mdx* (*n* = 6), and *mdx*-ApoE (*n* = 11). Mean + SEM. (PDF 176 kb)

Additional file 4 Figure S3. Animal body weights, heart weights, heart-body weight ratios, and cardiac histology. Body weight from 3 to 7 months on chow (A) and Western diets (B). Heart weight (C) and heart to body weight ratios (D) for groups at 7 months on Western diet. Examples of Masson’s trichrome stained hearts at 7 months of age on Western diet, scale bars 1 mm (left) and 250 μm (right) (E). Chow: WT (*n* = 3), ApoE (*n* = 5), *mdx* (*n* = 6), and *mdx*-ApoE (*n* = 11). Western: WT (*n* = 9), ApoE (*n* = 9), *mdx* (*n* = 5–11), and *mdx*-ApoE (*n* = 5–11). Mean ± SEM. *P < 0.05. (PDF 394 kb)

Additional file 5: Figure S4. Gastrocnemius muscle size and composition at 4 months on Western diet. Quantification of total gastrocnemius area in cm^2^ (A) and percentage of area composed of fat (B), fibrosis (C) and healthy myofiber (D). WT (*n* = 3), ApoE (*n* = 3), *mdx* (*n* = 6), and *mdx*-ApoE (*n* = 6). Mean + SEM. *P < 0.05 **P < 0.01 ***P < 0.001 ****P < 0.0001. (PDF 158 kb)

Additional file 6: Figure S5. Quadriceps femoris muscle size and composition at 4 and 7 months on Western diet. Representative images of quadriceps femoris of Western diet-fed 7-month-old mice stained with Masson’s trichrome, scale bars 500 μm (left) and 100 μm (right) (A). Quantification of total rectus femoris area in cm^2^ (B) and percentage of area composed of fat (C), fibrosis (D) and healthy myofiber (E). WT 4 m (*n* = 4), 7 m (*n* = 10); ApoE 4 m (*n* = 4), 7 m (*n* = 9); *mdx* 4 m (*n* = 7), 7 m (*n* = 7); *mdx*-ApoE 4 m (*n* = 6), 7 m (*n* = 11). Mean + SEM. *P < 0.05 **P < 0.01 ***P < 0.001 ****P < 0.0001. (PDF 346 kb)

Additional file 7: Figure S6. Diaphragm muscle size and composition at 4 and 7 months on Western diet. Representative images of diaphragm muscle of Western diet-fed 7-month-old mice stained with Masson’s trichrome, scale bars 500 μm (left) and 100 μm (right) (A). Quantification of total diaphragm area in square centimeter (B) and percentage of area composed of fat (C), fibrosis (D), and healthy myofiber (E). WT 4 m (*n* = 4), 7 m (*n* = 9); ApoE 4 m (*n* = 4), 7 m (*n* = 10); *mdx* 4 m (*n* = 7), 7 m (*n* = 7); *mdx*-ApoE 4 m (*n* = 6), 7 m (*n* = 11). Mean + SEM. *P < 0.05 **P < 0.01 ****P < 0.0001. (PDF 300 kb)

Additional file 8: Figure S7. Triceps brachii vascular density in areas of damage at 7 months on Western diet. Representative images of H&E and CD31 (PECAM-1) via IHC of *mdx* and *mdx*-ApoE in triceps brachii serial sections in areas of muscle damage, scale bars 200 μm. (PDF 308 kb)

## Abbreviations

ANOVA: Analysis of variance
ApoE: Apolipoprotein E
ARB: Angiotensin II type 1 receptor blocker
BMD: Becker muscular dystrophy
CK: Creatine kinase
DCM: Dilated cardiomyopathy
DGC: Dystrophin-associated glycoprotein complex
DKO: Double knockout
DMD: Duchenne muscular dystrophy
EDL: Extensor digitorum longus
h: Hour(s)
H&E: Hematoxylin and eosin
HDL: High-density lipoprotein
IDL: Intermediate-density lipoprotein
IHC: Immunohistochemistry
KO: Knockout
LDL: Low-density lipoprotein
m: Month(s)
MD: Muscular dystrophy
min: Minute(s)
NO: Nitric oxide
OCT: Optimal cutting temperature
SEM: Standard error of the mean
TA: Tibialis anterior
TG: Triglyceride
VLDL: Very low-density lipoprotein
WT: Wild-type
