# Supplementary material for: Increased plasma lipid levels exacerbate muscle pathology in the mdx mouse model of Duchenne muscular dystrophy
Source: Skelet Muscle. 2017 Sep 12;7:19. doi: 10.1186/s13395-017-0135-9 (PMC5596936; doi:10.1186/s13395-017-0135-9)
Supplement: Supplementary file 1 — Muscle and cardiac data stratified at 7 months on Western diet stratified by gender. WT (n = 5M, 4F); ApoE (n = 5M, 4F); mdx (n = 4M, 3F); and mdx-ApoE (n = 6M, 4F). Mean (SEM), P < 0.05 in bold. (PDF 434 kb) [file 13395_2017_135_MOESM1_ESM.pdf]

Supplementary Figure 8

Data by Gender: 7 Month Western Diet

		WT	ApoE	*mdx*	*mdx* -ApoE
Gastrocnemius					
Total Area	Male	0.244(0.016)	0.286(0.009)	0.208(0.012)	0.183(0.010)
	Female	0.225(0.018)	0.232(0.008)	0.229(0.022)	0.166(0.021)
	P	0.8634	0.0805	0.8604	0.8922
% Fat	Male	1.257(0.727)	0.060(0.041)	1.363(0.319)	14.411(3.288)
	Female	0.690(0.231)	0.239(0.073)	1.684(0.727)	19.678(2.363)
	P	0.9996	0.9999	0.9999	0.3349
% Fibrosis	Male	3.475(0.217)	3.341(0.267)	7.835(0.676)	25.466(2.647)
	Female	3.360(0.177)	4.166(0.699)	8.301(0.488)	30.250(4.766)
	P	0.9999	0.9977	0.9998	0.3601
% Myofiber	Male	98.741(0.729)	99.928(0.038)	97.801(0.983)	79.262(3.380)
	Female	99.310(0.231)	99.758(0.070)	96.503(0.674)	70.486(8.497)
	P	0.9999	0.9999	0.9976	0.1655
Triceps brachii					
Total Area	Male	0.166(0.009)	0.168(0.011)	0.179(0.009)	0.169(0014)
	Female	0.133(0.003)	0.136(0.007)	0.192(0008)	0.136(0.002)
	P	0.2662	0.239	0.9463	0.9694
% Fat	Male	2.591(0.298)	1.449(0.078)	1.993(0.334)	9.001(1.919)
	Female	2.863(0.323)	0.847(0.154)	1.936(0.392)	17.640(6.149)
	P	0.9999	0.9991	0.9999	0.1055
% Fibrosis	Male	4.414(0.455)	5.135(0.506)	6.440(0.904)	19.334(1.792)
	Female	3.597(0.106)	3.592(0.068)	9.221(1.018)	22.547(2.931)
	P	0.9896	0.8829	0.5926	0.2393
% Myofiber	Male	9.7414(0.292)	98.542(0.075)	96.418(0.895)	81.282(2.759)
	Female	97.137(0.323)	99.153(0.154)	95.668(0.723)	76.417(6.831)
	P	0.9999	0.9993	0.9991	0.1798

		WT	ApoE	*mdx*	*mdx* -ApoE
Heart					
Weight	Male	194.35(10.119)	185.85(16.045)	162.5(18.2)	163.23(15.295)
	Female	150.7(11.116)	146.92(4.337)	134.3(12.52)	171.15(2.750)
	P	**0.0444**	0.0634	0.539	0.9917
CO (ml/min)	Male	17.497(0.839)	17.752(3.055)	14.021(1.185)	17.028(1.001)
	Female	16.777(1.115)	15.607(0.736)	14.076(0.629)	17.022(1.340)
	P	0.9955	0.7553	0.9999	0.9976
EF (%)	Male	56.921(1.817)	54.561(0.919)	54.759(2.791)	53.712(1.227)
	Female	54.321(2.277)	55.947(0.805)	54.435(0.138)	52.58.2(2.367)
	P	0.8032	0.9686	0.9999	0.9768
FS (%)	Male	29.511(1.113)	28.062(0.631)	28.017(1.805)	27.516(0.765)
	Female	27.893(1.474)	28.757(0.461)	27.720(0.042)	26.751(1.469)
	P	0.8098	0.9864	0.9996	0.9699
SV (ml)	Male	41.557(0.608)	43.123(6.361)	36.078(2.961)	43.632(2.463)
	Female	40.196(1.605)	36.250(1.154)	34.736(2.284)	39.667(2.517)
	P	0.9975	0.4389	0.9976	0.7899
LVDD (mm)	Male	4.210(0.052)	4.190(0.245)	3.890(0.065)	4.253(0.116)
	Female	3.964(0.073)	3.876(0.075)	3.843(0.108)	4.128(0.157)
	P	0.6944	0.4176	0.9991	0.9283
LVSD (mm)	Male	3.0827(0.064)	3.013(0.160)	2.798(0.054)	3.085(0.101)
	Female	2.752(0.053)	2.765(0.071)	2.778(0.079)	3.028(0.165)
	P	0.2748	0.48	0.9999	0.992
LVDV (ml)	Male	79.135(2.300)	78.969(11.048)	65.696(2.616)	81.634(5.143)
	Female	68.697(3.035)	65.187(3.053)	63.858(4.314)	76.013(6.933)
	P	0.7218	0.4201	0.9994	0.9232
LVSV (ml)	Male	37.578(1.909)	35.846(4.751)	29618(1.350)	38.002(3.057)
	Female	28.501(1.430)	38.937(1.942)	29.122(2.030)	36.345(4.760)
	P	0.3376	0.5333	0.9999	0.9926

Expressed as mean(SEM). Abbreviations - M: male, F: Female, WT: wild-type, CO: cardiac output, EF: ejection fraction, FS: fractional shortening, SV: stroke volume, LV: left ventricle, LVDD: LV diastolic diameter, LVSD: LV systolic diameter, LVDV: LV diastolic volume, LVSV: LV systolic volume
