# Supplementary figures and images for: Increased plasma lipid levels exacerbate muscle pathology in the mdx mouse model of Duchenne muscular dystrophy
Source: Skelet Muscle. 2017 Sep 12;7:19. doi: 10.1186/s13395-017-0135-9 (PMC5596936; doi:10.1186/s13395-017-0135-9)

## A 4m Western Diet

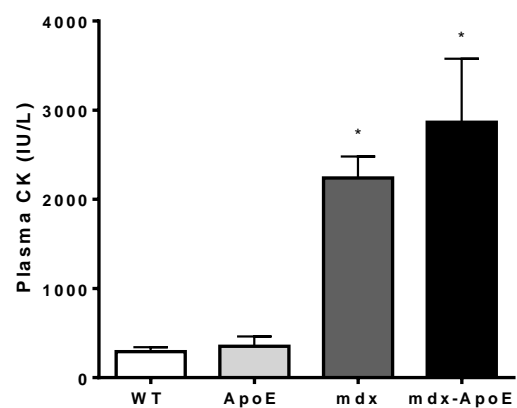

## B

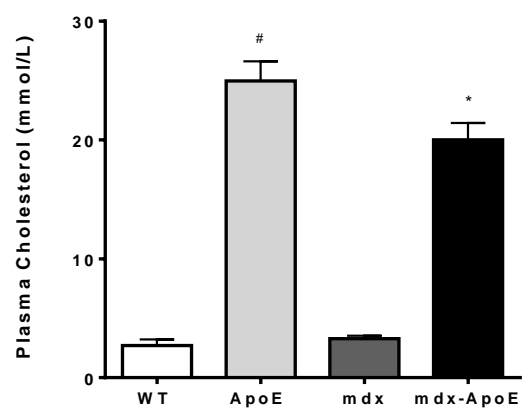

## C

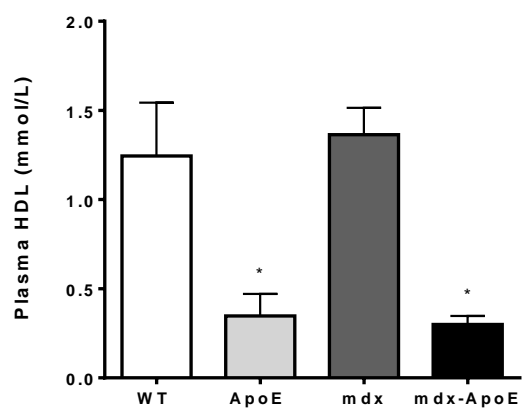

## D

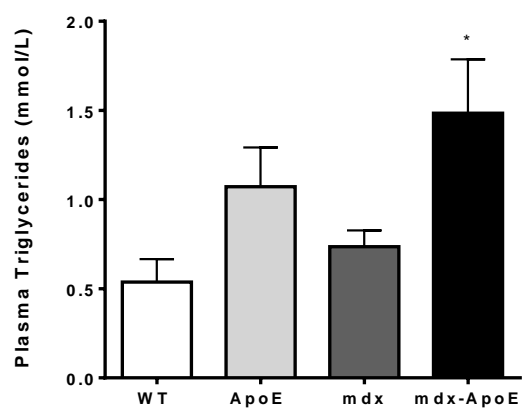

Supplement: Supplementary file 2 — Plasma lipid levels at 4 months of age on Western diet. Plasma CK in IU/L (A), total cholesterol in mmol/L (B), HDL in mmol/L (C) and TG in mmol/L (D) at 4 months of age on Western diet. WT (n = 4), ApoE (n = 4), mdx (n = 7), and mdx-ApoE (n = 5). Mean + SEM. *P < 0.05 compared to WT and mdx #P < 0.05 compared to all other groups. (PDF 158 kb) [file 13395_2017_135_MOESM2_ESM.pdf]

**A**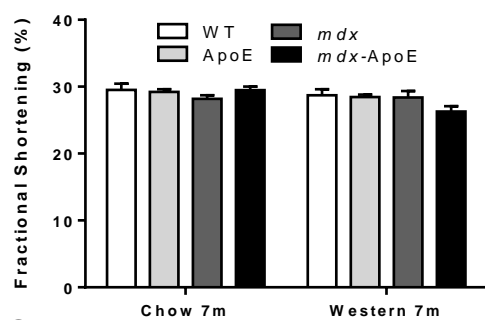**B**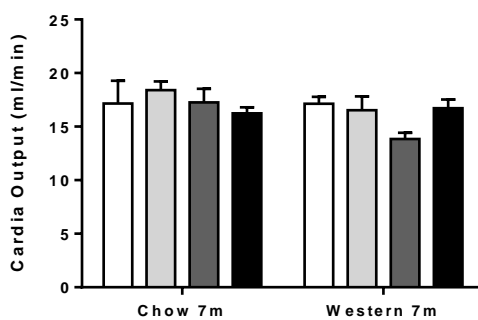**C**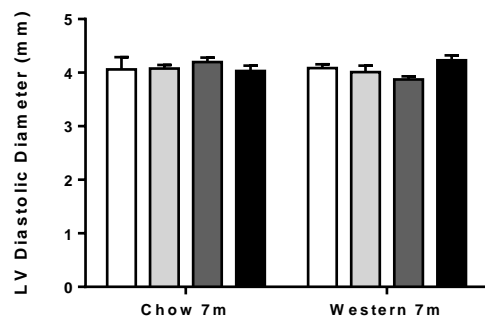**D**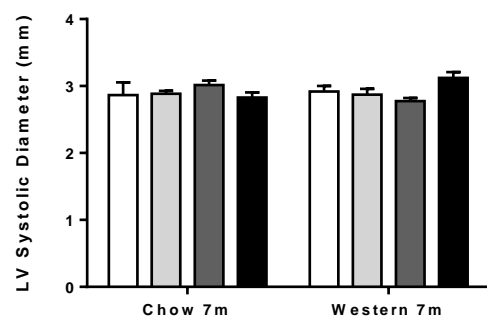**E**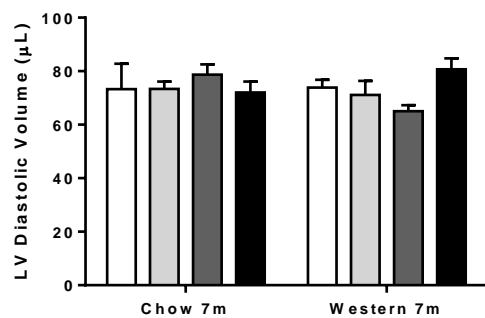**F**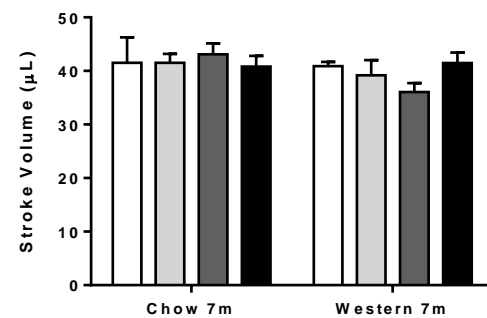

Supplement: Supplementary file 3 — Heart function parameters on chow and Western diets. Percent fractional shortening (A), cardiac output in mL/min (B), left ventricular diastolic diameter (C) and systolic diameter in mm (D), left ventricular diastolic volume (E) and stroke volume in μL (F). Chow: WT (n = 3), ApoE (n = 5), mdx (n = 6), and mdx-ApoE (n = 11). Western: WT (n = 6), ApoE (n = 7), mdx (n = 6), and mdx-ApoE (n = 11). Mean + SEM. (PDF 176 kb) [file 13395_2017_135_MOESM3_ESM.pdf]

## A Chow Diet

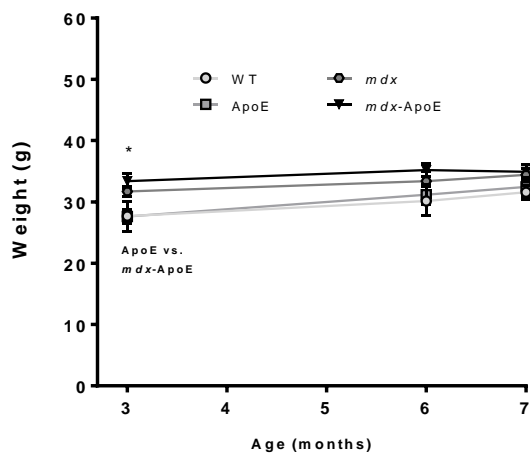

## B Western Diet

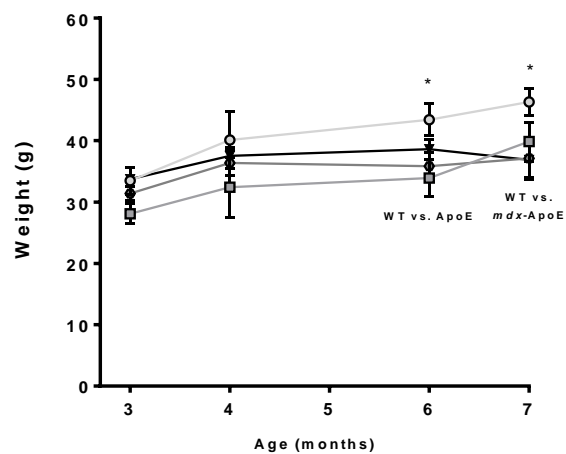

## C 7m Western Diet

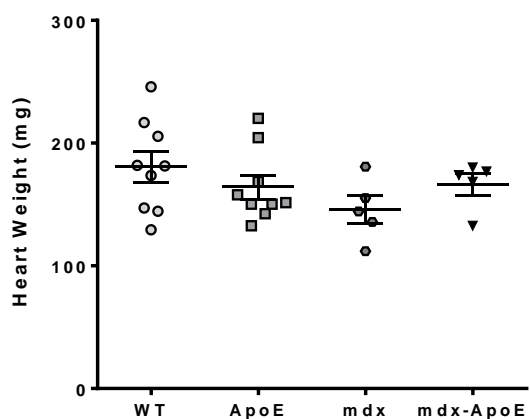

## D 7m Western Diet

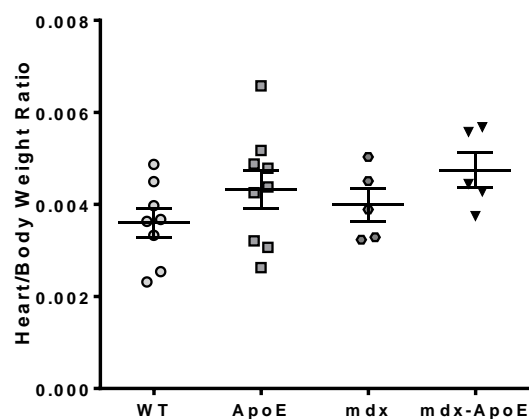

## E 7m Western Diet

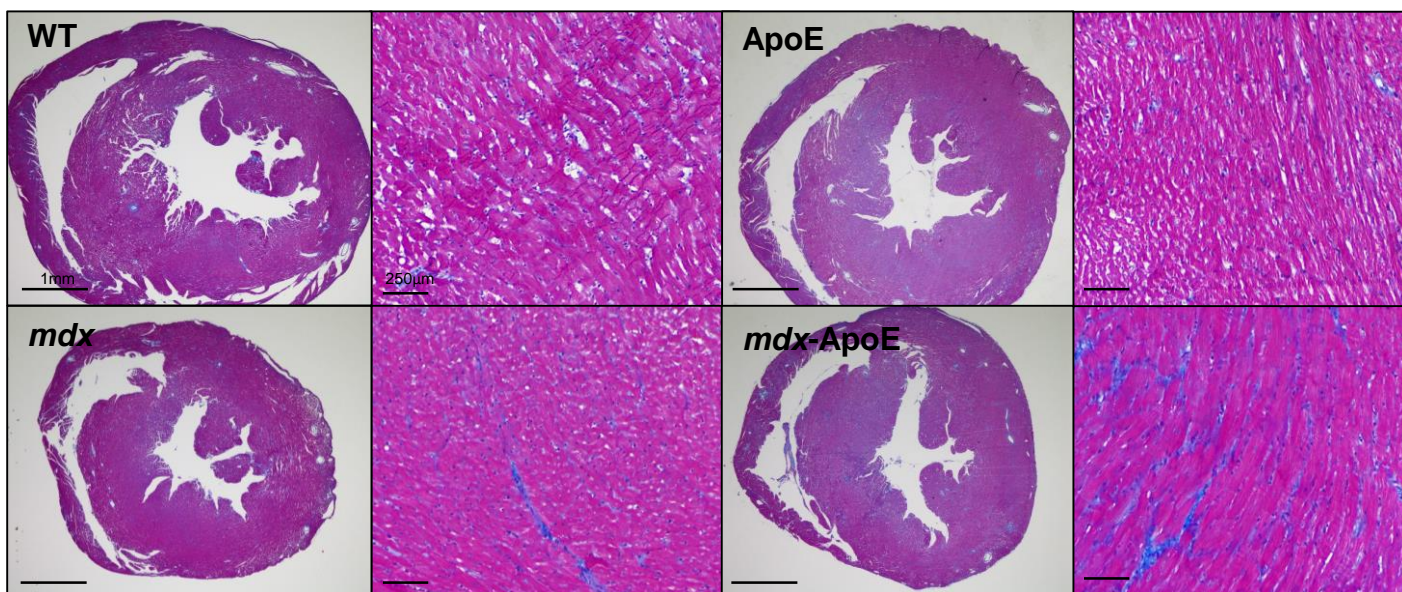

Supplement: Supplementary file 4 — Figure S3. Animal body weights, heart weights, heart-body weight ratios, and cardiac histology. Body weight from 3 to 7 months on chow (A) and Western diets (B). Heart weight (C) and heart to body weight ratios (D) for groups at 7 months on Western diet. Examples of Masson’s trichrome stained hearts at 7 months of age on Western diet, scale bars 1 mm (left) and 250 μm (right) (E). Chow: WT (n = 3), ApoE (n = 5), mdx (n = 6), and mdx-ApoE (n = 11). Western: WT (n = 9), ApoE (n = 9), mdx (n = 5–11), and mdx-ApoE (n = 5–11). Mean ± SEM. *P < 0.05. (PDF 394 kb) [file 13395_2017_135_MOESM4_ESM.pdf]

## A 4m Western Diet

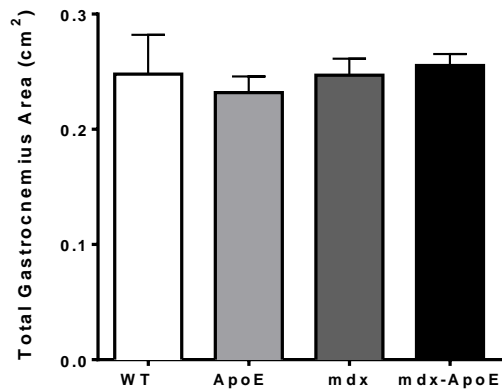

## B

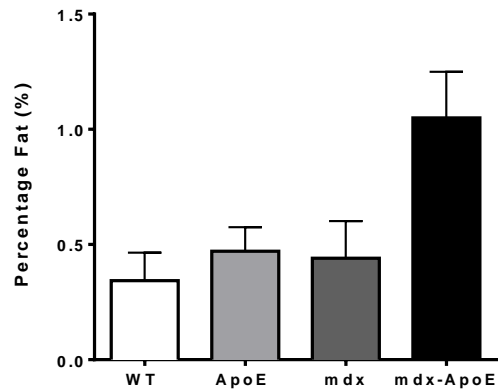

## C

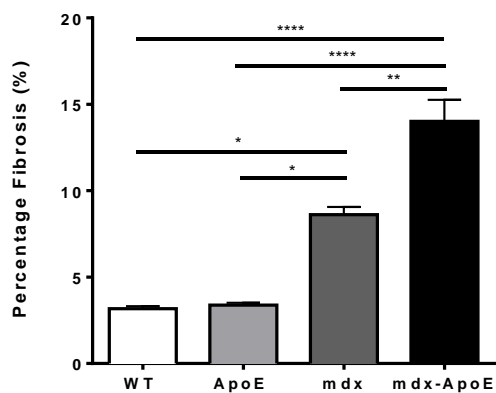

## D

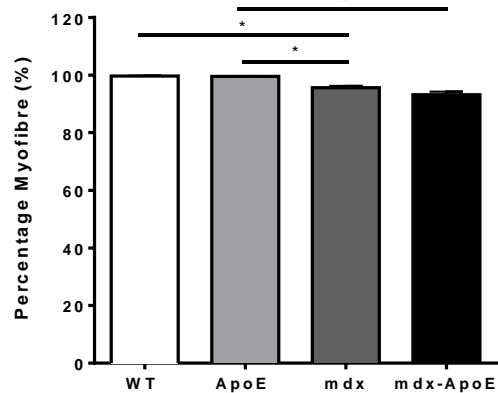

Supplement: Supplementary file 5 — Gastrocnemius muscle size and composition at 4 months on Western diet. Quantification of total gastrocnemius area in cm2 (A) and percentage of area composed of fat (B), fibrosis (C) and healthy myofiber (D). WT (n = 3), ApoE (n = 3), mdx (n = 6), and mdx-ApoE (n = 6). Mean + SEM. *P < 0.05 **P < 0.01 ***P < 0.001 ****P < 0.0001. (PDF 158 kb) [file 13395_2017_135_MOESM5_ESM.pdf]

A Western Diet 7m

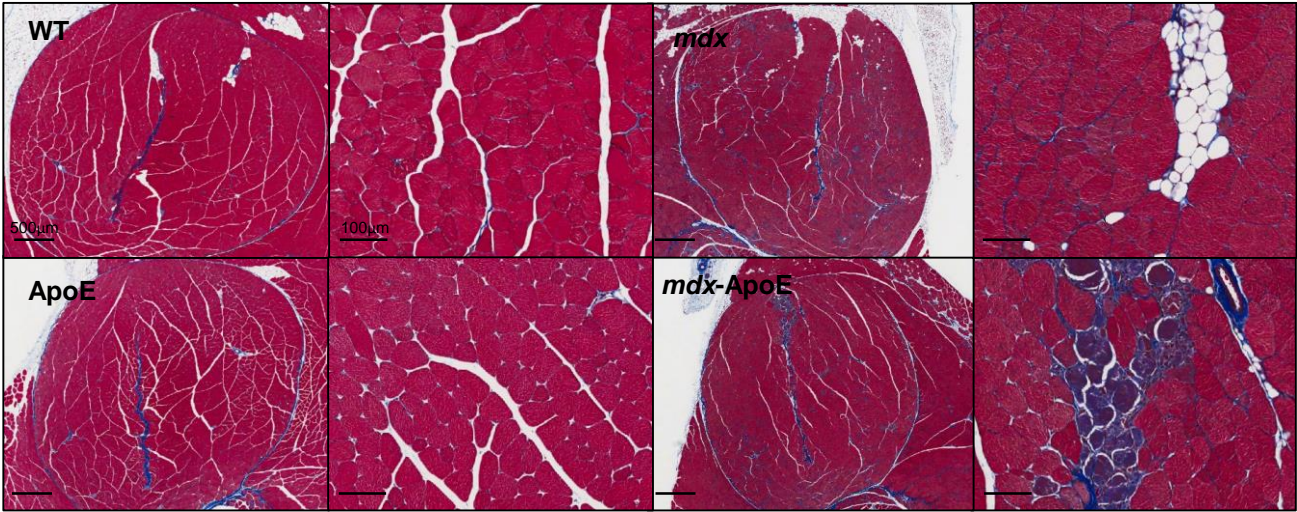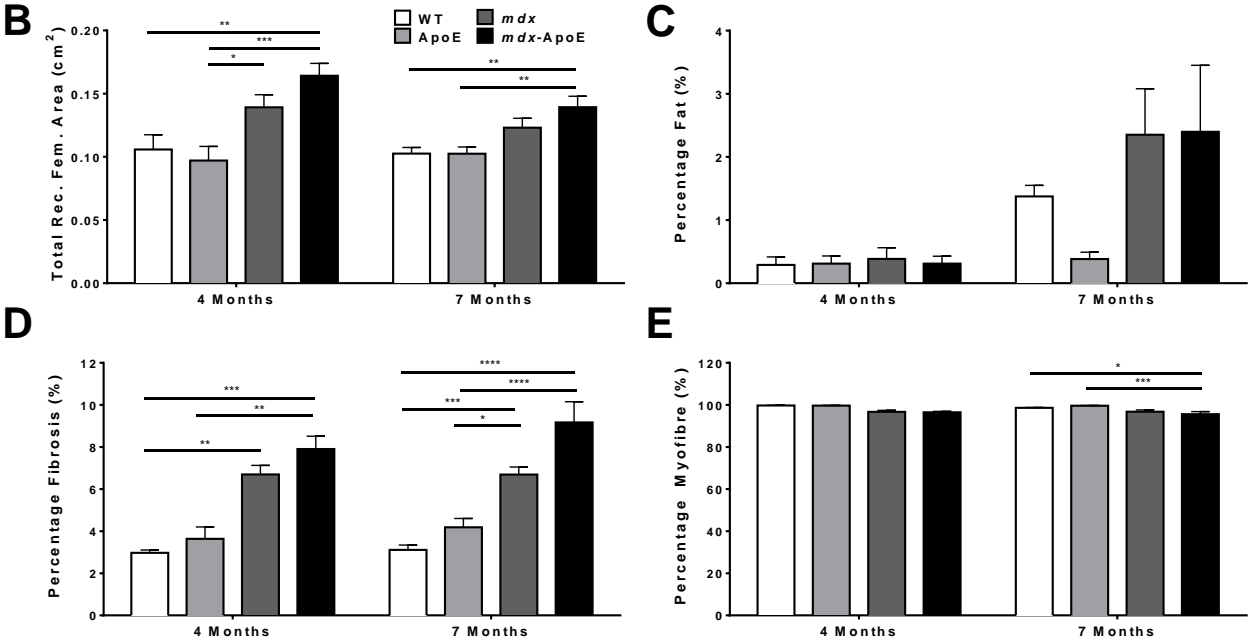

Supplement: Supplementary file 6 — Quadriceps femoris muscle size and composition at 4 and 7 months on Western diet. Representative images of quadriceps femoris of Western diet-fed 7-month-old mice stained with Masson’s trichrome, scale bars 500 μm (left) and 100 μm (right) (A). Quantification of total rectus femoris area in cm2 (B) and percentage of area composed of fat (C), fibrosis (D) and healthy myofiber (E). WT 4 m (n = 4), 7 m (n = 10); ApoE 4 m (n = 4), 7 m (n = 9); mdx 4 m (n = 7), 7 m (n = 7); mdx-ApoE 4 m (n = 6), 7 m (n = 11). Mean + SEM. *P < 0.05 **P < 0.01 ***P < 0.001 ****P < 0.0001. (PDF 346 kb) [file 13395_2017_135_MOESM6_ESM.pdf]

## A Western Diet 7m

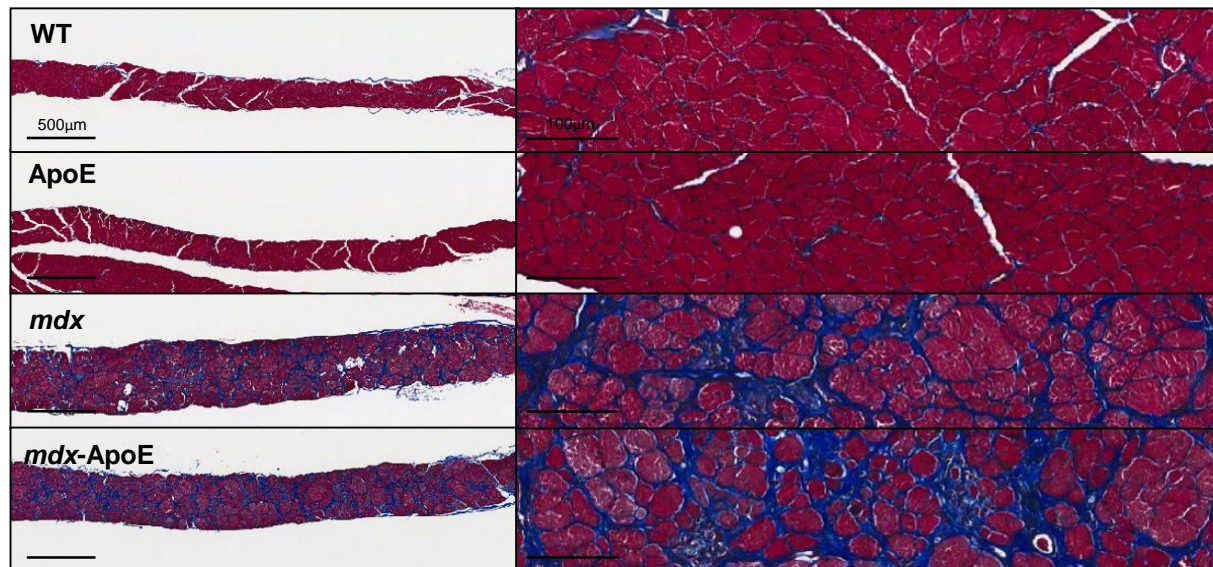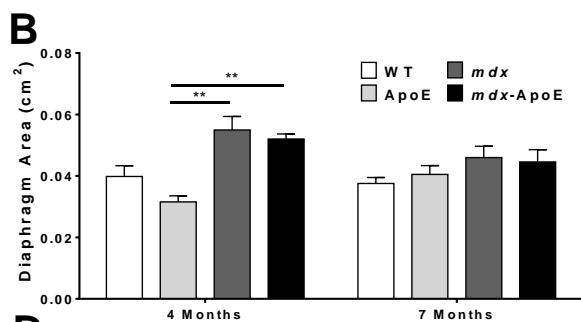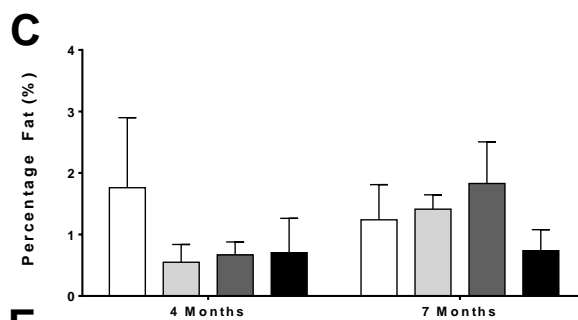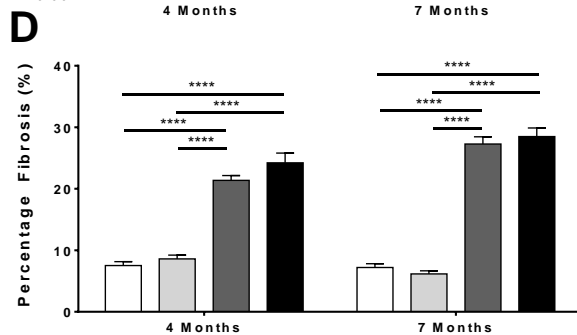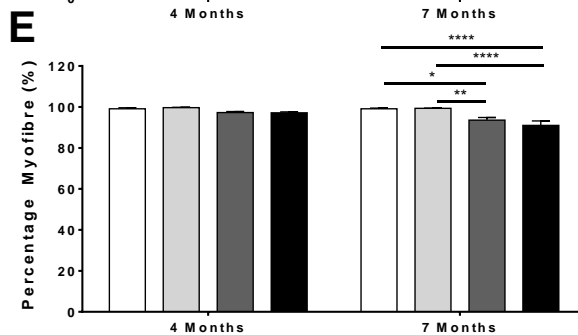

Supplement: Supplementary file 7 — Diaphragm muscle size and composition at 4 and 7 months on Western diet. Representative images of diaphragm muscle of Western diet-fed 7-month-old mice stained with Masson’s trichrome, scale bars 500 μm (left) and 100 μm (right) (A). Quantification of total diaphragm area in square centimeter (B) and percentage of area composed of fat (C), fibrosis (D), and healthy myofiber (E). WT 4 m (n = 4), 7 m (n = 9); ApoE 4 m (n = 4), 7 m (n = 10); mdx 4 m (n = 7), 7 m (n = 7); mdx-ApoE 4 m (n = 6), 7 m (n = 11). Mean + SEM. *P < 0.05 **P < 0.01 ****P < 0.0001. (PDF 300 kb) [file 13395_2017_135_MOESM7_ESM.pdf]

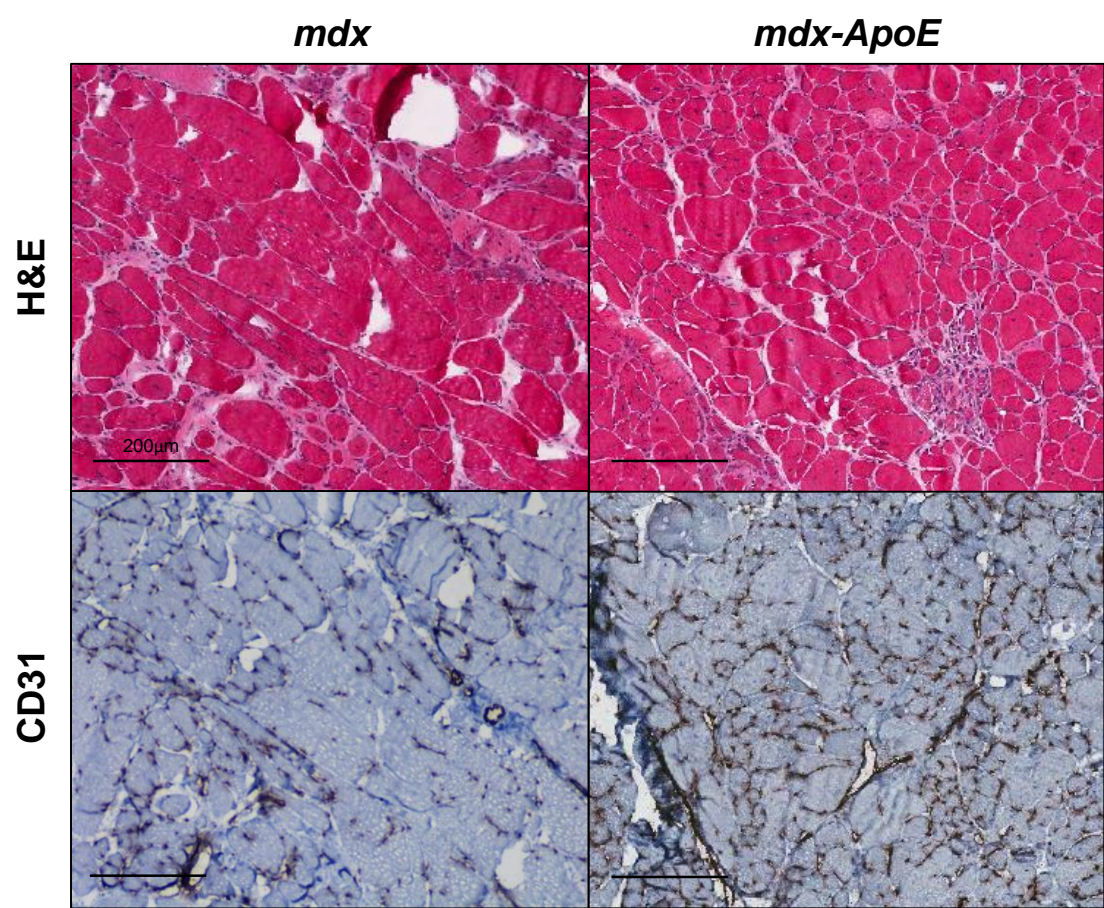

Supplementary Figure 7

Supplement: Supplementary file 8 — Triceps brachii vascular density in areas of damage at 7 months on Western diet. Representative images of H&E and CD31 (PECAM-1) via IHC of mdx and mdx-ApoE in triceps brachii serial sections in areas of muscle damage, scale bars 200 μm. (PDF 308 kb) [file 13395_2017_135_MOESM8_ESM.pdf]
